# RDPNet: A Multi-Scale Residual Dilated Pyramid Network with Entropy-Based Feature Fusion for Epileptic EEG Classification

**DOI:** 10.3390/e27080830

**Published:** 2025-08-05

**Authors:** Tongle Xie, Wei Zhao, Yanyouyou Liu, Shixiao Xiao

**Affiliations:** Big Data Analytics Laboratory, Chengyi College, Jimei University, Xiamen 361021, China; 202241013094@jmu.edu.cn (T.X.); 202241013093@jmu.edu.cn (Y.L.); xiaoshixiao@jmu.edu.cn (S.X.)

**Keywords:** epileptic seizure detection, deep learning, differential entropy, residual network, dilated convolution

## Abstract

Epilepsy is a prevalent neurological disorder affecting approximately 50 million individuals worldwide. Electroencephalogram (EEG) signals play a vital role in the diagnosis and analysis of epileptic seizures. However, traditional machine learning techniques often rely on handcrafted features, limiting their robustness and generalizability across diverse EEG acquisition settings, seizure types, and patients. To address these limitations, we propose RDPNet, a multi-scale residual dilated pyramid network with entropy-guided feature fusion for automated epileptic EEG classification. RDPNet combines residual convolution modules to extract local features and a dilated convolutional pyramid to capture long-range temporal dependencies. A dual-pathway fusion strategy integrates pooled and entropy-based features from both shallow and deep branches, enabling robust representation of spatial saliency and statistical complexity. We evaluate RDPNet on two benchmark datasets: the University of Bonn and TUSZ. On the Bonn dataset, RDPNet achieves 99.56–100% accuracy in binary classification, 99.29–99.79% in ternary tasks, and 95.10% in five-class classification. On the clinically realistic TUSZ dataset, it reaches a weighted F_1_-score of 95.72% across seven seizure types. Compared with several baselines, RDPNet consistently outperforms existing approaches, demonstrating superior robustness, generalizability, and clinical potential for epileptic EEG analysis.

## 1. Introduction

Epilepsy, a common neurological disorder affecting approximately 50 million individuals globally [1], imposes a substantial burden on patients’ quality of life and represents a significant socioeconomic challenge. The accurate diagnosis and classification of epilepsy types are paramount for the formulation of effective therapeutic strategies. Electroencephalography (EEG) serves as a cornerstone in the diagnostic toolkit for epilepsy and has been extensively adopted in clinical practice. Traditionally, neurologists rely on visual inspection of EEG recordings to identify epileptic events. Although expert-based interpretation remains the clinical gold standard, it is time-consuming, labor-intensive, and subject to inter-observer variability. The visual inspection of EEG signals by neurologists remains the clinical gold standard for epilepsy diagnosis, but it is time-consuming, labor-intensive, and prone to subjective variability.

To alleviate this burden, numerous automated seizure detection methods based on traditional machine learning have been proposed [2]. These methodologies primarily integrate conventional signal processing with machine learning techniques, focusing on the two critical stages of feature extraction and classification. In the feature extraction phase, investigators typically select EEG signal features manually, guided by empirical knowledge and observational data. Features derived from time, frequency, and time–frequency domains are extensively utilized for seizure identification. For instance, Sharmila and Geethanjali [3] pioneered the application of a combination of time-domain features, initially developed for electromyography signal analysis, such as waveform length, number of zero-crossings, and number of slope sign changes, to the detection of epileptic EEG signals. Wen and Zhang [4] proposed a frequency-domain feature selection method that combines sample entropy with a genetic algorithm for multi-class EEG signal analysis. Kambarova et al. [5] explored the utility of nonlinear dynamic methods, such as fractal dimension, in the analysis of electroencephalograms from healthy individuals and patients with epilepsy. AlSharabi et al. [6] introduced a diagnostic methodology for epilepsy founded on Discrete Wavelet Transform time–frequency analysis and Shannon entropy, which entails the decomposition of EEG signals into multiple time–frequency sub-bands followed by the extraction of entropy features. Building upon entropy-based approaches, Zhang et al. [7] proposed an automatic epileptic EEG classification approach based on differential entropy and attention mechanism. Their method decomposed EEG recordings into five sub-frequency bands and employed an improved attention model framework as the classifier for nonpatient-specific evaluation. Similarly, Akter et al. [8] developed a multi-band entropy-based feature extraction method focusing on high-frequency components (ripple and fast ripple) from interictal iEEG. They utilized eight different entropy measures, including approximate entropy, permutation entropy, Shannon entropy, sample entropy, Tsallis entropy, phase entropy, and Reny’s entropy, combined with sparse linear discriminant analysis for feature selection in epileptic focus identification. Entropy-based methods in epilepsy detection typically employ entropy measures such as input features [7,8], which are then fed into conventional machine learning classifiers. In addition to the aforementioned approaches, various nonlinear features have also been extensively applied to seizure detection. Madan et al. [9] investigated the application of the Hurst exponent, derived from the Discrete Wavelet Transform, in epilepsy detection.

The classification stage typically employs a variety of machine learning classifiers to identify epileptic seizure activity. Guo et al. [10] utilized artificial neural networks in conjunction with waveform complexity metrics to achieve automated detection of epileptic seizures EEGs. Brinkmann et al. [11] applied support vector machine algorithms to analyze intracranial EEG data, successfully forecasting naturally occurring seizures. Wang et al. [12] enhanced the recognition accuracy of multi-level epileptic states by combining random forests with grid search optimization. Na et al. [13] improved the accuracy of epilepsy diagnosis by integrating an extended K-nearest neighbors classifier with a multi-distance decision-making mechanism. Although the aforementioned methods have shown good performance in certain epilepsy classification tasks, they still face two major limitations. First, they rely on handcrafted feature extraction based on domain expertise, which may fail to capture complex nonlinear features in EEG signals [14,15]. Second, their generalization ability is often limited, making it difficult to adapt to new datasets or heterogeneous patient populations [16].

To address the limitations of traditional machine learning methods, recent studies have increasingly turned to deep learning for EEG decoding. Deep learning models support end-to-end learning directly from raw or minimally preprocessed EEG signals, eliminating the need for handcrafted features and reducing the subjectivity of feature engineering [17,18,19,20,21]. Compared to traditional methods, deep learning offers more robust and consistent diagnostic support for clinicians, as well as improved generalizability across diverse patient populations and clinical settings for patients.

Currently, Convolutional Neural Networks (CNNs) and Recurrent Neural Networks (RNNs) represent the most frequently employed deep learning models in epilepsy detection. CNNs have demonstrated significant advantages in this domain, primarily through their capacity to automatically extract high-level features from EEG signals, thereby circumventing the limitations of manual feature engineering inherent in traditional machine learning and enabling end-to-end automated diagnostic workflows. By leveraging multiple convolutional and pooling layers, CNNs can effectively learn intricate patterns and temporal dependencies within EEG signals. However, this methodology is confronted with challenges, including substantial requirements for training data, high computational complexity, and a propensity for overfitting when data are scarce. Moreover, the inherent constraints imposed by CNN kernel sizes often impede the capture of long-range dependencies crucial in EEG time-series analysis. In contrast to CNNs, which are predominantly oriented towards spatial feature extraction, RNNs, as deep learning architectures expressly tailored for time-series data, exhibit distinct advantages in epilepsy detection. They are particularly well suited for processing physiological data such as EEG signals, which are characterized by temporal dependencies and variable lengths. Long Short-Term Memory (LSTM) networks, a specialized RNN variant, effectively address the short-term memory and vanishing gradient problems of conventional RNNs through sophisticated gating mechanisms, enabling the capture of long-term temporal dependencies in EEG signals. Gated Recurrent Units (GRUs), another RNN variant, offer reduced model complexity by consolidating gating structures while maintaining robust sequence modeling capabilities. The inherent architecture of RNNs is naturally suited for the detection and prediction of time-series events like epileptic seizures, as they can learn complex temporal patterns within EEG signals. Nevertheless, RNN-based approaches also possess limitations, including protracted training durations, sensitivity to sequence length, high computational demands, and potential susceptibility to gradient-related issues when processing long sequences [22]. Beyond CNNs and RNNs, recent studies have explored alternative deep learning paradigms tailored to the unique dynamics of brain signals. Li et al. [23] proposed a graph-generative network to dynamically model evolving brain connectivity patterns. Ghosh et al. [24] introduced a deep oscillatory neural network that uses Hopf oscillators to capture frequency-specific oscillatory dynamics. Similarly, Hadad et al. [25] employed a biologically inspired spiking neural network to decode cognitive states from EEG signals using event-driven spike processing.

Despite significant advancements in deep learning methodologies, several key challenges persist in automated epileptic EEG classification. Existing models often struggle to simultaneously capture local detail features and long-range temporal dependencies in EEG signals effectively, while traditional entropy-based features are predominantly used as static inputs, failing to dynamically characterize the statistical complexity of deep network activations. Additionally, multi-scale feature fusion processes frequently encounter issues such as scale mismatch and redundant information interference. To address these challenges, we propose RDPNet, a novel multi-scale Residual Dilated Pyramid Network, with the following key contributions:

(1) We designed a novel network architecture for seizure detection by integrating residual convolutional module and dilated convolutional pyramids to jointly capture local features and global temporal dependencies in EEG signals.
(2) We introduced an entropy-guided dual-pathway fusion strategy that combined global max pooling with dynamically computed differential entropy features, enhancing the discriminability and statistical robustness of multi-scale representations.
(3) We conducted comprehensive experiments on the University of Bonn and Temple University Hospital EEG Seizure Corpus (TUSZ) benchmark datasets, and we demonstrated that RDPNet consistently outperformed several baseline methods in terms of classification accuracy and generalization across diverse clinical scenarios.

The remainder of this paper is organized as follows: Section 2 presents related research and the current state of epilepsy detection technology. Section 3 details the research methodology and experimental results, encompassing an introduction to the dataset, data preprocessing procedures, the architectural design of the proposed RDPNet along with its constituent components, ten-fold cross-validation results, ablation studies, parameter sensitivity analysis, feature visualization, and comprehensive comparative evaluation with baseline methods. Section 4 discusses the limitations of the study and future research directions. Section 5 summarizes the main contributions and findings of the entire paper. Through these sections, we will comprehensively demonstrate the efficacy of the proposed model and its prospective applications in the field of epilepsy detection.

## 2. Related Work

In the domain of neural network-based epilepsy classification, deep learning methodologies have demonstrated substantial developmental potential. Initial research endeavors predominantly focused on the application of foundational CNNs. Acharya et al. [26] pioneered the application of deep convolutional neural networks to seizure detection in EEG signals, proposing an end-to-end automated analysis framework capable of autonomously learning and identifying features requisite for classification directly from raw EEG data. Truong et al. [27] introduced a CNN-based method for seizure prediction, which extracts critical feature information through time-frequency domain transformation of EEG signals; this approach can automatically generate optimized features for each patient to optimally classify preictal and interictal segments. The method exhibits robust universality and generalization capabilities, consequently lowering the implementation threshold and expertise prerequisites for epilepsy prediction technologies. Pachori and Gandhi [28] proposed a methodology integrating Fourier-Bessel Series Expansion (FBSE) with CNN classification for epileptic seizure detection. Their approach decomposes EEG signals into five rhythmic components using FBSE, applies Euclidean distance metrics to generate image representations, and subsequently classifies these images through a CNN architecture. This work demonstrates the potential of combining traditional signal processing techniques with deep learning frameworks for seizure detection. Zhao et al. [29] designed a one-dimensional CNN employing larger convolutional kernels for seizure detection, constructing an end-to-end architecture comprising three convolutional blocks and three fully connected layers. This model integrates batch normalization and dropout layers within conventional convolutional blocks to augment model learning capacity and prevent overfitting.

Although foundational CNN models have demonstrated promising results in epilepsy detection, they continue to face limitations when handling more complex EEG signals, particularly in capturing multi-scale and long-range temporal patterns. To address these challenges, researchers have explored advanced CNN variants, notably Residual Networks (ResNets) and dilated convolutions, which offer improved representational capacity and feature extraction capabilities. ResNets have been introduced to mitigate the degradation and vanishing gradient problems associated with deep networks. For instance, Gao et al. [30] employed a ResNet152 architecture for epileptic EEG classification. In their approach, EEG signals were first transformed into power spectral density energy diagrams, and the residual blocks of ResNet152 were leveraged to extract deep features. In parallel, to effectively expand the receptive field for capturing long-range dependencies in time-series data without incurring additional computational costs, dilated convolutions have been introduced into the field of seizure prediction. Hussein et al. [31] proposed a novel “semi-dilated convolution” module, which mapped EEG signals to two-dimensional wavelet scalograms and applied dilated convolutions to handle the resulting nonsquare inputs, thereby improving seizure prediction accuracy. Building on this idea, Gao et al. [32] further developed a spatiotemporal multi-scale convolutional network that utilizes dilated convolutions with different dilation rates in parallel across both temporal and spatial dimensions. This design enables the aggregation of multi-scale information from local to global levels, significantly enhancing patient-specific seizure prediction performance.

Although CNNs and their variants are effective in extracting spatial features, they are limited in modeling the temporal dependencies inherent in EEG signals. To address this, RNNs and their advanced variants, such as LSTM and GRU networks, have been widely adopted due to their ability to capture sequential patterns and mitigate gradient-related issues in long time-series data. Tsiouris et al. [33] employed LSTM networks for long-term seizure prediction using continuous EEG recordings spanning several hours. Their approach incorporated features from both time and frequency domains, as well as interchannel correlations and graph-theoretic measures, across varying prediction windows from 15 min to 2 h. Zhang et al. [34] proposed a seizure detection method based on a Bidirectional GRU (BiGRU) network, which captures both forward and backward temporal dependencies to improve classification performance. Similarly, Najafi et al. [35] developed an RNN-LSTM-based model to distinguish between focal and generalized epilepsy, leveraging time–frequency features with a focus on theta-band activity.

As research has progressed, investigators have recognized the limitations of singular network architectures, leading to the development of more robust hybrid frameworks that amalgamate the spatial feature extraction capabilities of CNNs with the temporal modeling strengths of RNNs. Roy et al. [36] introduced ChronoNet, a specialized RNN-based architecture for abnormal EEG detection. ChronoNet integrates stacked one-dimensional convolutional layers with deep GRU layers. Xu et al. [37] developed a one-dimensional CNN-LSTM model for epileptic seizure recognition, wherein a CNN effectively extracts features from normalized EEG sequence data, which are subsequently processed by LSTM layers to further extract temporal characteristics. Zhao et al. [38] introduced the ResBiLSTM hybrid deep learning method; this model initially employs a one-dimensional ResNet to adeptly extract local spatial features from EEG signals, following which the acquired features are input into a bidirectional Long Short-Term Memory (BiLSTM) network layer to model temporal dependencies, achieving end-to-end seizure detection through the integration of three residual blocks and the BiLSTM network. Moreover, Sun et al. [39] proposed a causal spatiotemporal model that integrates transfer entropy-based causal graphs with GAT and BiLSTM to enhance epileptic seizure detection by capturing both interchannel causal relationships and spatiotemporal dynamics. Despite these advancements, the nonlinear structure and multi-scale temporal characteristics of EEG signals continue to pose challenges for accurate seizure detection. Drawing on the strengths of residual connections, dilated convolutions, and entropy-based measures, we designed a hybrid architecture to improve automated epileptic EEG classification.

## 3. Materials and Methods

### 3.1. The Benchmark Datasets

#### 3.1.1. The Bonn Dataset

**Figure 1:**
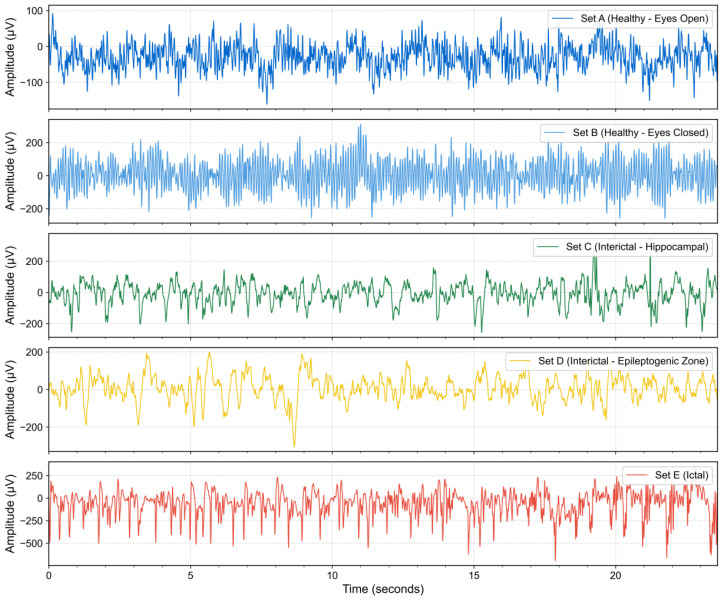
EEG signal examples from the five categories (A–E) in the Bonn dataset.

The Bonn dataset is a widely used EEG benchmark in epilepsy research, provided by the University of Bonn, as acquired by Andrzejak et al. [40]. The data have been rigorously screened and preprocessed by clinical specialists, with muscle artifacts and other interfering signals removed, ensuring a high signal-to-noise ratio and providing a standard reference for evaluating machine learning and deep learning methods in epilepsy detection. This dataset is based on EEG recordings from 10 subjects, including 5 healthy volunteers and 5 diagnosed epilepsy patients. Data acquisition followed the international standard 10–20 electrode placement system with a sampling frequency of 173.61 Hz. The complete dataset is divided into five distinct categories, designated as categories A, B, C, D, and E, each containing 100 single-channel EEG recordings with a duration of 23.6 s, generating 4097 sampling points per segment. Figure 1 shows example EEG signals from the five categories.

Sets A and B were collected from healthy volunteers, representing normal EEG activity during eyes-open and eyes-closed states, respectively. Sets C and D recorded interictal EEG signals from epilepsy patients, with Set C originating from brain regions contralateral to the epileptogenic zone, while Set D was recorded from the epileptogenic zone. Set E recorded ictal EEG signals.

#### 3.1.2. The TUSZ Dataset

The TUSZ dataset stands as one of the largest and most well-acknowledged open-source epilepsy EEG datasets available to researchers, offering detailed clinical case descriptions [41]. It includes annotations on the timing and types of epileptic seizures, as well as comprehensive patient information such as sex, age, medications, clinical history, seizure event count, and duration. Our study utilized the May 2020 release of the corpus (V1.5.2), comprising 3050 seizure cases across eight distinct seizure types, recorded at various sampling frequencies and montages. The seizure types include Focal Non-Specific Seizure (FNSZ), Generalized Non-Specific Seizure (GNSZ), Absence Seizure (ABSZ), Complex Partial Seizure (CPSZ), Tonic Clonic Seizure (TCSZ), Tonic Seizure (TNSZ), Simple Partial Seizure (SPSZ), and Myoclonic Seizure (MYSZ). Due to the limited number of MYSZ events, we excluded this type and focused on the remaining seven seizure categories for analysis.

### 3.2. Data Preprocessing

To ensure adequate model training and further enhance model robustness, this study first performed data segmentation, then applied Gaussian white noise perturbation to the segmented EEG signals. Each complete EEG signal (4097 sampling points) was equally divided into 8 nonoverlapping consecutive subsequences, with each subsequence containing 512 sampling points. Formally, given an original signal $S=\{s_{1},s_{2},\ldots ,s_{4097}\}$, the nonoverlapping window strategy generated a set of subsequences $\left\{S_{1},S_{2},\ldots ,S_{8}\right\}$, where $S_{i}=\{s_{(i-1)\times 512+1},s_{(i-1)\times 512+2},\ldots ,s_{i\times 512}\}$, i∈{1,2,…,8}.

The augmented signals were generated according to

(1) x=S+α×σ×n

where *S* is the original EEG training signal, *n* is Gaussian noise with zero mean and unit variance, *σ* is the standard deviation of *S*, and *α* is a scaling factor that controls the noise intensity. In this study, *α* was set to 0.01, and the number of augmented samples was doubled relative to the original training set, following the optimal configuration validated in Zhao et al. [38].

In our experiments with the Bonn dataset, we generated twice the number of artificially augmented data for each original training sample. Furthermore, for the TUSZ dataset, we employed the IBM TUSZ data preparation version for building data [42], which utilizes the transverse central parietal montage featuring 20 selected paired channels as input. Additionally, all EEG recordings were resampled to a uniform frequency of 250 Hz.

### 3.3. RDPNet Model Architecture

#### 3.3.1. Overall Model Framework

**Figure 2:**
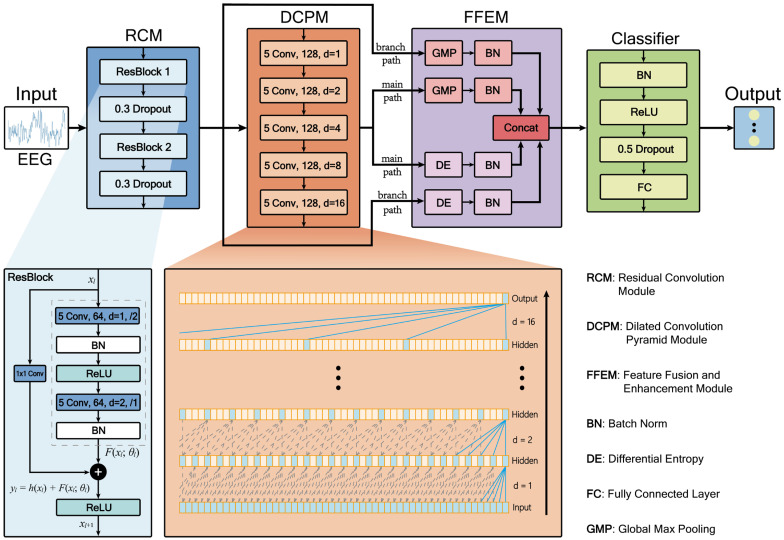
RDPNet model architecture.

This study proposes a hybrid architecture, RDPNet, for the automatic classification of epileptic EEG signals. RDPNet adopts an end-to-end learning framework that directly processes raw EEG data, eliminating the need for complex manual feature engineering typically required in traditional methods. As illustrated in Figure 2, the model comprises four key functional modules: the Residual Convolution Module (RCM), the Dilated Convolution Pyramid Module (DCPM), the Feature Fusion and Enhancement Module (FFEM), and the classifier.

The RCM extracts local features from EEG signals using two residual blocks (ResBlocks) while mitigating the vanishing gradient problem. The DCPM consists of five cascaded convolutional layers with varying dilation rates, enabling the network to capture long-range temporal dependencies by expanding the receptive field without increasing parameter count. The FFEM fuses pooled and entropy-based features from both the residual and dilated pathways, capturing both structural and statistical information. Finally, the classifier consists of a single fully connected (FC) layer that performs the final classification.

Figure 2 adopts commonly used shorthand notation to represent model parameters. For example, “5 Conv, 64, d = 1, /2” denotes a convolutional layer with a kernel size of 5, 64 output channels, a dilation rate of 1, and a stride of 2; “0.3 Dropout” indicates a dropout layer with a drop rate of 0.3.

#### 3.3.2. RCM

The RCM comprises two residual blocks, each followed by a dropout layer (drop rate 0.3) to mitigate overfitting. Each residual block contains two convolutional layers, as illustrated in Figure 2. In the first block, both convolutional layers have 64 filters. The first layer uses a stride of 2 for downsampling, followed by batch normalization (BN) and ReLU activation. The second layer employs a stride of 1 and a dilation rate of 2 to expand the temporal receptive field, also followed by BN. The second residual block follows a similar structure but uses 128 filters. Each residual block includes a main path and a shortcut connection. Due to the mismatch in input and output dimensions, the shortcut path applies a 1 × 1 convolution for dimensional alignment. Finally, the outputs of the main and shortcut paths are combined via element-wise addition and passed through a ReLU activation to produce the final output of the residual block. The mathematical expression of the residual block can be formalized as

(2) $$\begin{array}{c} y_{l}=h(x_{l})+F(x_{l};\theta_{l}) \end{array}$$

(3) $$\begin{array}{c} x_{l+1}=f(y_{l}) \end{array}$$

where $x_{l}$ and $x_{l+1}$ represent the input and output of the l-th residual block, respectively. F is a residual function, $\theta_{l}$ includes all learnable parameters in the l-th residual block, and f is a ReLU activation function. In our design, $h(x_{l})$ is a convolutional function (1 × 1 convolution for dimension matching), and the residual function $F(x_{l};\theta_{l})$ contains two convolutional layers.

#### 3.3.3. DCPM

Dilated convolution expands the receptive field by setting dilation rates, effectively capturing long-range temporal dependencies without increasing parameter count and computational complexity. As shown in Figure 2, the dilated convolution pyramid module in RDPNet adopts a cascaded structure consisting of 5 consecutive dilated convolutional layers designed to capture long-range dependencies. The module takes feature maps from residual blocks as input, with each layer using dilated convolution with kernel size 5, followed by BN and ReLU activation. The dilation rates of these 5 layers increase exponentially: 1, 2, 4, 8, and 16.

Dilated convolution can be mathematically expressed as

(4) $$\begin{array}{c} F\left(t\right)=\sum_{i=0}^{k-1}W\left(i\right)\cdot x\left(t-d\cdot i\right) \end{array}$$

where F(t) is the output feature, W(i) represents convolution kernel weights, x(t) is the input feature, k is the kernel size, and d is the dilation rate. This design allows the model to capture temporal dependencies over multiple scales, which is particularly beneficial for modeling complex patterns in epileptic EEG signals.

For cascaded dilated convolutions, the receptive field (RF) can be calculated using

(5) $$RF=1+\sum_{i=1}^{n}(k_{i}-1)\times d_{i}$$

where n is the number of layers, $k_{i}$ denotes the kernel size, and $d_{i}$ represents the dilation rate of the i-th convolutional layer.

#### 3.3.4. FFEM

The FFEM adopts a dual-pathway strategy to integrate shallow features from the residual convolution module and deep features from the dilated convolutional pyramid. Unlike prior methods that merely concatenate feature maps, FFEM extracts two complementary representations: global max pooling features that capture salient local activations across channels and entropy-based features computed via differential entropy to reflect the statistical complexity of feature distributions.

A key contribution of FFEM lies in its differentiated processing and enhancement of multi-scale features. Differential entropy is computed separately for the shallow and deep feature maps to generate statistical representations. To address distribution mismatch, the module applies independent batch normalization to four distinct feature types: pooled and entropy features from both the main and branch pathways.

These four vectors are subsequently concatenated to form a unified 512-dimensional representation that effectively integrates spatial saliency and statistical complexity. To compute the differential entropy of the feature map, we first calculate the unbiased variance along the temporal dimension. Then, the differential entropy is computed as

(6) $$H_{diff}=\frac{1}{2}\log(2\pi e(\sigma^{2}+\epsilon ))$$

where $\sigma^{2}$ is the unbiased variance across the temporal dimension, and ϵ is a small constant for numerical stability.

#### 3.3.5. Classifier

The classifier first applies BN and ReLU activation to the input features from FFEM, followed by a dropout layer with a rate of 0.5 for regularization, and finally outputs class logits through a fully connected layer. Final classification is achieved through the softmax function, which converts the FC layer outputs into a probability distribution defined as follows:

(7) $$P(y=j|x)=\frac{e^{z_{j}}}{\sum_{k=1}^{C}e^{z_{k}}}$$

where $z_{j}$ is the logit corresponding to class *j*, and *C* is the total number of classes. The softmax function converts the logits into a probability distribution over all classes, ensuring that the output probabilities sum to 1 and enabling multi-class classification.

Additionally, this study employs cross-entropy loss with label smoothing as the optimization objective. The loss function with label smoothing is defined as

(8) $$L_{ls}=-\frac{1}{N}\sum_{i=1}^{N}\sum_{j=1}^{C}{\tilde{y}}_{i,j}\log(p_{i,j})$$

where $L_{ls}$ denotes the cross-entropy loss with label smoothing, N is the batch size, C is the number of classes, ${\tilde{y}}_{i,j}$ represents the smoothed labels, and $p_{i,j}$ is the predicted probability output by the softmax function. To improve model generalization, we introduce label smoothing which converts hard labels to soft labels:

(9) $${\tilde{y}}_{i,j}=(1-\alpha )y_{i,j}+\frac{\alpha }{C}$$

where α is the label smoothing coefficient, set to 0.2 in this study, and $y_{i,j}$ represents the one-hot-encoded ground truth labels. Label smoothing mitigates model overconfidence by softening the target distribution, thereby enhancing generalization performance.

#### 3.3.6. Experimental Configuration

All experiments were conducted on an NVIDIA RTX 4090 GPU using the PyTorch framework (version 2.2.2). The model was trained with the Adam optimizer using an initial learning rate of 0.0005, a weight decay coefficient of *λ* = 0.0001, a batch size of 64, and a maximum of 100 training epochs. To improve training stability, gradient clipping with a maximum norm of *γ* = 1.0 was applied to prevent gradient explosion, and early stopping was employed to mitigate overfitting.

Table 1 summarizes the network architecture and hyperparameter settings of RDPNet, which were determined through extensive empirical tuning.

Table 1 adopts commonly used shorthand notation to represent model parameters. For example, “5 Conv, 64, d = 1, /2” denotes a convolutional layer with a kernel size of 5, 64 output channels, a dilation rate of 1, and a stride of 2; “Dropout: 0.3” represents a dropout layer with a drop rate of 0.3. In the DCPM, convolutional layers with progressively increasing dilation rates (d = 1, 2, 4, 8, 16) were employed to construct a cascaded feature extraction structure. The FFEM was used to extract global features through global max pooling and differential entropy for each branch pathway, which were then concatenated to form a 512-dimensional feature vector. The classifier employed a dropout layer with a drop rate of 0.5 and a linear layer for final classification output.

#### 3.3.7. Evaluation Methodology

Model evaluation was conducted using k-fold cross-validation to enhance statistical reliability and reduce evaluation bias. Model selection followed the “training set optimum” principle, selecting the model with the highest training accuracy and lowest training loss as the final model for testing. Evaluation metrics included Accuracy, Precision, Recall, and F_1_-score (F_1_).

To comprehensively assess the model’s adaptability across scenarios of varying complexity, we designed multi-level classification tasks for systematic evaluation. On the Bonn dataset, 13 classification tasks were designed, including 7 binary classification tasks (e.g., A-E, B-E, C-E), 5 ternary classification tasks (e.g., A-C-E, A-D-E, B-C-E), and 1 five-class classification task (A-B-C-D-E). On the TUSZ dataset, we performed a seven-class classification to identify distinct seizure types.

Given the severe class imbalance among the seven epileptic seizure types in the TUSZ dataset, weighted F_1_-score (F_1,w_) was employed for comprehensive evaluation, assigning weights proportional to each class’s sample size to effectively mitigate the impact of class imbalance on evaluation results.

### 3.4. Experimental Results

Table 2 presents the ten-fold cross-validation performance of RDPNet across 13 classification tasks on the Bonn dataset. Binary tasks achieved near-perfect results, with mean accuracies ranging from 99.56% to 100%. Ternary tasks consistently maintained high performance, with mean accuracies between 99.29% and 99.79%. For the more challenging five-class task, RDPNet achieved a mean accuracy of 95.10%. Across all tasks, accuracies remained consistently high across all ten folds (k1–k10), indicating strong generalization and architectural stability.

### 3.5. Ablation Studies

To comprehensively evaluate the effectiveness of key components in the RDPNet model, this study designed and executed a series of ablation experiments. By selectively removing critical components from the model, we quantified the contribution of each component to overall performance. This study conducted 3 ablation experiments:

- Experiment w/o RCM: The RCM was removed, and the EEG signal was fed directly into the DCPM for feature extraction. This setting evaluates the contribution of RCM to feature learning.
- Experiment w/o DCPM: The DCPM was removed, and the output of the RCM was directly connected to the classifier. This experiment assesses the importance of progressive receptive field expansion and long-range temporal dependency modeling.
- Experiment w/o entropy features: Entropy-based features in the FFEM were removed, and only pooled features were used for classification. This configuration quantifies the contribution of entropy information to classification performance.

#### 3.5.1. Component Contribution Analysis

As shown in Table 3, ablation experiments were conducted to evaluate the individual contributions of each RDPNet module to the overall classification performance. Removing the RCM led to a 1.95% drop in accuracy (from 95.10% to 93.15%) in the five-class task, indicating its effectiveness in alleviating gradient vanishing and facilitating the learning of meaningful representations. Eliminating the DCPM caused the most substantial degradation, with accuracy falling to 85.10%, reflecting a 9.90% decrease. This highlights the importance of progressive receptive field expansion in modeling long-range temporal dependencies in epileptic EEG signals. Excluding the entropy-based features resulted in a 0.88% decrease in accuracy, underscoring their complementary role in capturing statistical complexity. The incorporation of differential entropy facilitates uncertainty modeling over feature distributions, thus improving the model’s capacity to discriminate between epileptic states. The ablated models also exhibited varying degrees of decline in Precision, Recall, and F_1_-score.

#### 3.5.2. Classification Confusion Matrix Analysis

**Figure 3:**
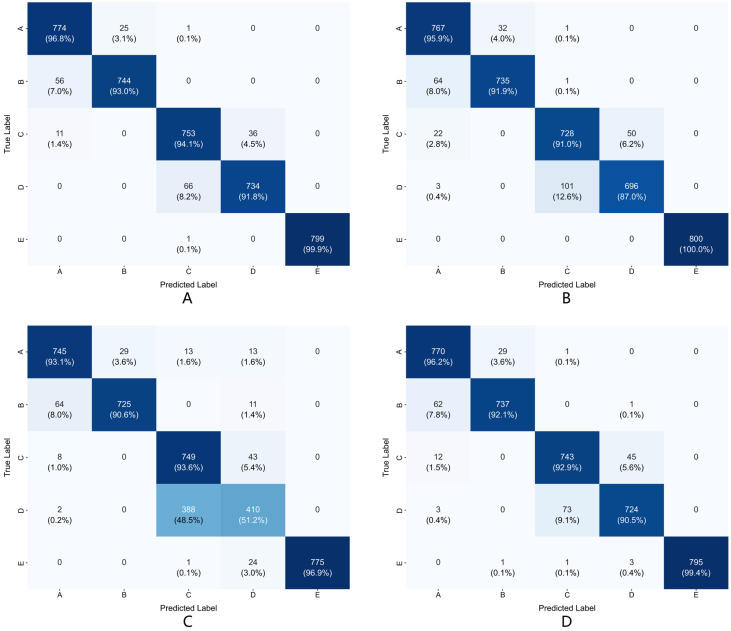
Confusion matrices of different model configurations in the five-category classification task: (**A**) complete model; (**B**) w/o RCM; (**C**) w/o DCPM; (**D**) w/o entropy features.

Figure 3 presents the confusion matrices of different ablated model configurations in the five-class task. As shown in Figure 3A, the complete model demonstrated excellent classification performance across all categories. Figure 3B shows that removing the residual convolution module caused relatively moderate performance degradation, mainly manifested as slight increases in misclassification between various categories. Figure 3C reveals that removing the dilated convolution pyramid module caused the most severe classification confusion, particularly resulting in significant misclassification between classes C and D, indicating that the dilated convolution pyramid module is crucial for distinguishing interictal signals from different brain regions. Figure 3D demonstrates that while removing the differential entropy calculation of feature maps caused relatively modest performance degradation, it still impacted the overall classification stability of the model, confirming the contribution of differential entropy based on statistical complexity of feature distributions in enhancing classification robustness.

### 3.6. Hyperparameters Sensitivity Analysis

The performance of deep learning models is highly sensitive to hyperparameter settings. Proper configuration is essential for optimizing model behavior and ensuring practical applicability. To identify the optimal parameter settings for RDPNet and assess the impact of key hyperparameters on classification performance, we conducted a systematic sensitivity analysis. By varying critical architectural parameters and observing their effects, this study provides both theoretical insights and empirical guidance for effective model configuration.

#### 3.6.1. Impact of Convolution Kernel Sizes

This study investigated the effect of different kernel size combinations used in the residual blocks of the RCM and the dilated convolutions of the DCPM on classification performance, as summarized in Table 4. The findings reveal a nonlinear relationship between kernel size and model accuracy. For the residual blocks, a kernel size of 5 yielded the best performance. This size appears well suited for capturing local temporal features in EEG signals while avoiding excessive noise or feature distortion. In contrast, smaller kernels (e.g., size 3) restricted the receptive field, limiting the model’s ability to extract informative patterns. Larger kernels (e.g., size 7) may have introduced over-smoothing, reducing sensitivity to key epileptic features. Similarly, for dilated convolutions, a kernel size of 5 also led to the best results. This setting effectively balances receptive field expansion and feature resolution, aligning well with the temporal characteristics of epileptic EEG activity. While dilated convolutions are designed to capture long-range dependencies, overly large kernels may dilute critical temporal detail. The combination of kernel size 5 in both residual and dilated convolution modules achieved optimal performance, demonstrating a synergistic balance between local feature extraction and long-range temporal modeling. These results underscore the importance of carefully configuring kernel sizes when designing architectures for epileptic EEG classification.

#### 3.6.2. Impact of Entropy Types

**Figure 4:**
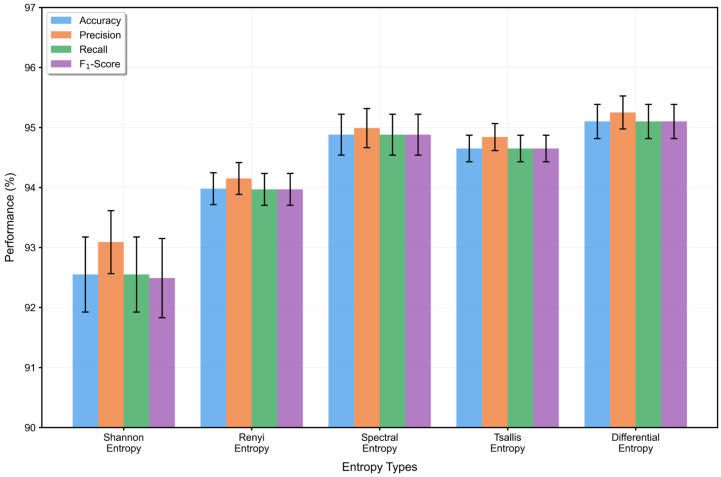
Impact of different entropy feature types on five-class classification performance (%).

To evaluate the sensitivity of RDPNet to different entropy-based statistical descriptors, we conducted comparative experiments using five commonly used entropy measures: Shannon entropy, Renyi entropy, spectral entropy, Tsallis entropy, and differential entropy. Except for the type of entropy used in the FFEM, all other network components remained unchanged. The results, shown in Figure 4, indicate notable differences in classification performance across entropy types. Shannon and Renyi entropy yielded lower accuracies (92.5–94.2%), possibly due to their reliance on discretizing continuous-valued feature maps and, in the case of Renyi entropy, the use of a fixed order parameter. These factors may limit their ability to flexibly characterize fine-grained or adaptive distributional variations in deep feature activations. Spectral and Tsallis entropy achieved moderate improvements (94.7–94.9%), leveraging frequency-domain structure and nonextensive statistical mechanics, respectively. However, spectral entropy may lose spatial context due to Fourier-based transformation, while the performance of Tsallis entropy is affected by parameter sensitivity, which may limit its generalization capability. Differential entropy achieved the best performance across all metrics, with both accuracy and F_1_-score reaching 95.1%. Unlike other entropies, it directly operates on continuous-valued features without requiring discretization or domain transformation. Its estimation via unbiased variance enables adaptive quantification of activation dispersion patterns under varying epileptic states, thereby providing highly discriminative statistical cues for classification. These findings underscore the critical role of entropy selection in statistical feature modeling and confirm that differential entropy provides the most effective and robust representation for epileptic EEG classification.

### 3.7. Feature Visualization

**Figure 5:**
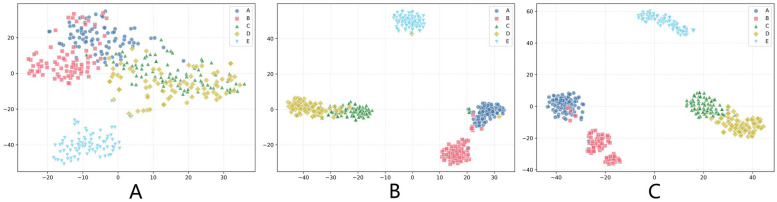
t-SNE feature visualization: (**A**) RCM output, (**B**) DCPM output, (**C**) complete model output.

t-SNE is a nonlinear dimensionality reduction technique that maps high-dimensional data to two- or three-dimensional space for visualization by preserving local similarities between data points, making it particularly suitable for revealing clustering structures and patterns in complex data. Figure 5 presents the t-SNE visualization results of feature representations at different RDPNet processing stages, clearly demonstrating the progressive improvement in feature separation capability.

As shown in Figure 5A, the output of the residual convolution module achieved good cluster separation for class E, but class C and class D exhibited complete overlap, while class A and class B also demonstrated substantial overlap, indicating that local features extracted solely by residual blocks have limitations in distinguishing classes with similar characteristics. After processing through the dilated convolution pyramid module, Figure 5B shows that class E maintained good separation, the intercluster distances between classes C and D significantly increased with markedly reduced cluster overlap, and the cluster separability of classes A and B also improved notably, validating the effectiveness of the dilated convolution pyramid in progressive receptive field expansion and long-range temporal dependency modeling. Figure 5C demonstrates the optimal separation effect of the complete model, where the dual-pathway feature fusion strategy integrated shallow features from residual blocks and deep features from the dilated convolution pyramid, combined with differential entropy statistical complexity information of feature maps, resulting in classes C and D having only slight overlap at cluster boundaries, with classes A and B also achieving high cluster separability, and all five classes forming more compact cluster structures with significantly improved intracluster compactness and clearer intercluster boundaries, validating the remarkable effectiveness of the multi-scale feature fusion and differential entropy feature calculation strategy in enhancing the model’s feature representation capability.

### 3.8. Comparative Evaluation with Baseline Methods

#### 3.8.1. Baseline Comparison on Bonn Dataset

This study conducted comprehensive experiments on 13 classification tasks derived from the University of Bonn epilepsy EEG dataset, comprising 7 binary classification tasks, 5 ternary classification tasks, and 1 five-class task. Table 5 presents the performance comparison between RDPNet and four recent deep learning baseline methods: ResNet + LSTM (Qiu et al., 2023 [43]), ReBiLSTM (Zhao et al., 2024 [38]), CNN-Bi-LSTM (Cao et al., 2025 [44]), and CNN + LSTM (Shanmugam & Dharmar, 2023 [45]).

We computed 95% confidence intervals (CIs) based on the t-distribution for RDPNet using ten-fold cross-validation results across each task. Although formal paired testing could not be applied due to the absence of fold-level baseline results, we considered any baseline whose reported mean fell below RDPNet’s CI lower bound to exhibit a statistically distinguishable difference under a conservative criterion (α = 0.05).

As demonstrated in Table 5, RDPNet delivered outstanding performance across nearly all tasks. In binary classification tasks, the method achieved 100% accuracy on the A-E task (tied with ReBiLSTM and ResNet + LSTM), attained 99.94% accuracy on the B-E task (exceeding ReBiLSTM’s 99.88%), and obtained optimal results on AB-E, CD-E, and ABCD-E tasks. In ternary classification tasks, RDPNet outperformed all baseline methods, with accuracies ranging from 99.29% to 99.79%. In the five-class task, the method reached 95.10% accuracy, surpassing CNN + LSTM (92.50%), ReBiLSTM (91.27%), and ResNet + LSTM (90.17%).

Although all competing methods achieved high accuracy on the Bonn dataset, our method demonstrated consistently superior performance under conservative evaluation conditions. Statistical analysis shows that the number of tasks where baseline methods fell below the lower bound of RDPNet’s 95% confidence interval (CI) were as follows: ReBiLSTM in 5 out of 13 tasks, ResNet + LSTM in 2 out of 6, CNN-Bi-LSTM in 8 out of 9, and CNN + LSTM in all 5 tasks. The consistently narrow CIs derived from ten-fold cross-validation further highlight RDPNet’s stability; notably, even the lowest single-fold accuracy in the five-class task exceeded the average accuracy of most baseline methods. These results collectively confirm the strong adaptability and robustness of RDPNet for automated epilepsy diagnosis.

#### 3.8.2. Baseline Comparison on TUSZ Dataset

This section presents benchmark results for seven-class epileptic-seizure-type classification on the clinically challenging TUSZ dataset. Table 6 compares RDPNet with seven baseline approaches: CE-stSENet (Li et al., 2020 [46]), VWCNNs (Jia et al., 2022 [47]), NLTWSVM (Zhang et al., 2022 [48]), GGN (Li et al., 2022 [23]), MHA-CNN (Gill et al., 2024 [49]), 3D-CBAMNet (Huang et al., 2023 [50]), and ResBiLSTM (Zhao et al., 2024 [38]). Using the five-fold cross-validation results of RDPNet, we computed t-distribution-based 95% CIs for both accuracy and weighted F_1_-score (Acc/F_1,w_ CI: 95.14–96.32%/95.13–96.31%). Any competing method whose point estimate fell below the lower bound was considered to exhibit statistical differences at a one-sided α = 0.05 level.

RDPNet attained 95.73% accuracy and 95.72% weighted F_1_-score. Under conservative judgment conditions, our method demonstrated superior performance compared to all baseline methods. In fact, the performance of all seven baseline methods fell below the lower bound of RDPNet’s 95% CI (7/7 statistical differences), confirming the statistical robustness of the improvement. The enhancement derives from three technical advantages: end-to-end representation learning—unlike traditional models such as NLTWSVM that rely on handcrafted features, RDPNet automatically extracts discriminative patterns directly from raw EEG; hybrid residual-dilated architecture—compared with pure CNN schemes (CE-stSENet, VWCNNs), the residual pathway stabilizes optimization, while the dilated convolution pyramid enlarges receptive fields cost-effectively; dual-pathway fusion mechanism—relative to attention models (MHA-CNN) or 3D convolutions (3D-CBAMNet), the dual-pathway fusion mechanism with differential entropy enhancement more efficiently captures long-range temporal dynamics. The results demonstrate that RDPNet generalizes well to real clinical data with severe class imbalance, providing a reliable tool for multi-class seizure diagnosis.

## 4. Discussion

This section presents a comprehensive discussion of the experimental findings of RDPNet, focusing on its architectural components, entropy-based feature modeling, parameter sensitivity, temporal window selection, computational efficiency, and interpretability through feature visualization.

Ablation studies confirm the importance of each module. Removing the RCM resulted in noticeable performance degradation, indicating that residual connections help alleviate gradient vanishing and facilitate the learning of deeper representations. This is particularly important for modeling the complex nonlinear characteristics of epileptic EEG signals. The removal of the DCPM led to the most significant decline in performance, emphasizing the critical role of multi-scale temporal modeling. By progressively expanding the receptive field, dilated convolutions allow the model to capture temporal dynamics without increasing the number of parameters, which is essential for recognizing seizure patterns. Furthermore, excluding the differential entropy features from the FFEM reduced the classification performance, suggesting that quantifying statistical uncertainty contributes additional discriminative information. This highlights the value of integrating information-theoretic descriptors to enhance the model’s ability to distinguish between different epileptic states.

Parameter sensitivity analysis of convolution kernel sizes validates the rationality of the model design, indicating that appropriate kernel size selection is crucial for capturing the feature scales of EEG signals. Experimental results show that the optimal combination of residual block and dilated convolution kernels achieves an effective balance between local feature extraction and long-range dependency modeling. This finding emphasizes the importance of considering signal-specific characteristics in deep learning architecture design, providing valuable guidance for parameter selection in physiological signal processing.

Table 7 reveals the critical impact of temporal window length selection on model performance. As window length increased from 1 s to 3 s, the five-class accuracy improved from 88.87% to a peak of 95.1%. When window length further extended to 4 s, accuracy decreased to 94.95%. The FLOPs increased linearly with window length, rising from 25.86 million operations for the 1-s window to 101.74 million operations for the 4-s window. The single-sample inference time increased slightly with window length, from 1.806 ms to 1.879 ms. The results indicate that the 3-s temporal window achieves the optimal balance between accuracy and computational overhead.

From an information-theoretic perspective, entropy serves as a fundamental measure of uncertainty and holds unique value in feature representation. While existing studies typically use entropy as handcrafted prior knowledge derived from raw EEG signals, our approach computes differential entropy on dual-pathway features processed through residual networks and dilated convolutional pyramids. Unlike traditional entropy metrics that rely on discretization, differential entropy operates directly on continuous-valued deep feature maps, allowing for a more accurate characterization of the statistical complexity and uncertainty embedded in learned representations.

Compared with existing methods, RDPNet offers pronounced technical advantages through its multi-scale hybrid architecture. Unlike CNN-LSTM pipelines that rely on sequential processing, RDPNet leverages residual and dilated convolutions to effectively model both short- and long-range temporal dependencies in EEG signals. The dual-pathway fusion mechanism further aggregates local and long-range cues, while FFEM introduces a differential entropy computation method for deep feature maps that dynamically computes differential entropy to quantify the statistical variability of deep feature maps, improving the recognition capability for subtle epileptic state differences. Due to the published baselines report only task-level ten-fold means and omit per-fold statistics, paired tests such as the Wilcoxon signed-rank could not be applied; we therefore adopted a conservative one-sided criterion (α = 0.05): a baseline is considered to perform worse when its mean accuracy falls below the lower bound of RDPNet’s t-based 95% confidence interval. Under this conservative evaluation criterion, RDPNet demonstrates superior performance compared to baseline methods on both binary and multi-class epileptic-EEG tasks; notably, on the severely imbalanced seven-class TUSZ benchmark, every competing method fell outside RDPNet’s CI (Acc: 95.14–96.32%; F_1,w_: 95.13–96.31%), indicating the model’s robustness and clinical relevance.

To address the concern regarding computational efficiency, we conducted a quantitative comparison between RDPNet and ReBiLSTM, one of the best-performing and publicly available baseline models, since most other baselines do not provide open-source implementations. RDPNet contains approximately 569 k parameters with a computational complexity of 75.27 M FLOPs, and it achieves an average inference time of 1.843 ms per sample. In contrast, ReBiLSTM has 315 k parameters, 45.99 M FLOPs, and an inference time of 1.231 ms.

Although RDPNet incurs a slightly higher computational cost, with an additional 0.61 milliseconds in inference time per sample, this overhead is considered acceptable and worthwhile in the context of critical clinical applications such as real-time epileptic seizure detection. Experimental results show that RDPNet achieved a 3.83% improvement in accuracy over ReBiLSTM on the five-class task of the Bonn dataset and a 0.7% gain on the seven-class classification task for different seizure types using the clinically sourced TUSZ dataset. These findings demonstrate that RDPNet offers a favorable balance between accuracy and efficiency, making it a strong candidate for deployment in medical scenarios that demand both high diagnostic precision and real-time responsiveness.

Feature visualization analysis provides intuitive evidence for the model’s effectiveness. Visualization results show that the model’s feature representation capability presents progressive improvement across different processing stages, particularly achieving effective separation for difficult-to-distinguish categories. This separation capability has important significance for clinical applications, as accurately distinguishing different epileptic states is the foundation for diagnosis and treatment decisions.

However, this study also has some limitations that need to be addressed in future work. First, the model’s generalization capability needs further validation on larger-scale and more diverse datasets. While benchmark datasets provide standardized platforms for model evaluation, signal characteristics in real clinical environments may be more complex and variable, including different acquisition devices, electrode configurations, and patient population characteristics. Second, the multi-module architecture incurs relatively high computational overhead, which may become a limiting factor in real-time monitoring applications. Third, while the cross-validation strategy employed in this study ensures statistical reliability, the lack of strict subject-independent validation may limit the conclusiveness of the model’s generalization performance assessment on unseen patients. Fourth, the model’s interpretability mechanisms need further enhancement to improve its acceptability and credibility in clinical practice. Medical applications have high requirements for the transparency of model decision-making processes, particularly requiring intuitive mechanisms for clinical reasoning explanations of individual predictions.

Future research can be extended in several directions. First, this can include developing stronger generalization techniques, such as domain adaptation and transfer learning methods, to improve the model’s applicability across different datasets and clinical environments. Cross-dataset validation and multi-center clinical trials will be important steps for validating the model’s practical utility. Second, the work can delve into exploring lightweight architectural designs to reduce computational complexity, making the model more suitable for deployment in resource-constrained environments, particularly portable monitoring devices. Third, conducting rigorous subject-independent evaluations using patient-level data splitting strategies to better assess the model’s generalization performance in real-world deployment scenarios will be necessary. Fourth, we can explore integrating interpretability mechanisms, such as Grad-CAM and other attention visualization and feature importance analysis methods, to enhance the transparency of the model’s decision-making process and promote its application in clinical practice. Meanwhile, combining domain knowledge and clinical experience to develop explanation frameworks that better align with medical diagnostic logic will help improve the model’s acceptance in clinical environments.

## 5. Conclusions

This study proposes RDPNet, a multi-scale residual dilated pyramid network with entropy-based feature fusion for automatic classification of epileptic EEG signals. The model extracts local features through residual blocks and captures long-range temporal dependencies via dilated convolution pyramid modules. To further enhance classification performance, a dual-pathway multi-scale feature fusion strategy is employed, incorporating differential entropy to quantify the statistical complexity of deep feature maps. This entropy-based enhancement provides additional discriminative information, enabling more accurate identification of epileptic patterns. Extensive experiments on 13 classification tasks using the Bonn dataset demonstrate that RDPNet achieved 99.56–100% accuracy in binary classification tasks, 99.29–99.79% accuracy in ternary classification tasks, and 95.10% accuracy in the most challenging five-class task, demonstrating superior performance compared to baseline methods. On the TUSZ dataset, the model achieved a weighted F_1_-score of 95.72%, outperforming all baseline methods and demonstrating robustness on imbalanced datasets. Ablation experiments and parameter sensitivity analysis further validated the effectiveness of each component and the rationality of the model design. Feature visualization analysis provides intuitive evidence for the model’s feature representation capability. These results demonstrate RDPNet’s technical advantages in the field of epilepsy detection, providing an efficient and reliable solution for clinical EEG automatic analysis with significant clinical application value.

## Figures and Tables

**Table 1:** Layer-wise configuration and dimensionality of RDPNet.

Module	Configuration	Output Shape
ResBlock 1	5 Conv, 64, d = 1, /2	[B, 64, T/2]
	5 Conv, 64, d = 2, /1	
	Dropout: 0.3	
ResBlock 2	5 Conv, 128, d = 1, /2	[B, 128, T/4]
	5 Conv, 128, d = 2, /1	
	Dropout: 0.3	
Dilated Convolution Pyramid Module (DCPM)	5 Conv, 128, d = 1, /1	[B, 128, T/4]
	5 Conv, 128, d =2, /1	
	5 Conv, 128, d = 4, /1	
	5 Conv, 128, d = 8, /1	
	5 Conv, 128, d = 16, /1	
Feature Fusion and Enhancement Module (FFEM)	Global Max Pooling × 2	[B, 256]
	Differential Entropy × 2	[B, 256]
	Concat	[B, 512]
Classifier	Dropout: 0.5	[B, C]
	Linear C	

Note: B = Batch size; T = Time steps; C = Number of classes.

**Table 2:** Ten-fold CV accuracy (%) and overall evaluation metrics of the RDPNet model on Bonn dataset.

Dataset		k1	k2	k3	k4	k5	k6	k7	k8	k9	k10	Mean
**Binary**	**A-E**	100	100	100	100	100	100	100	100	100	100	**100**
	**B-E**	100	100	100	100	100	99.38	100	100	100	100	**99.94**
	**C-E**	100	100	100	100	100	100	99.38	99.38	100	100	**99.88**
	**D-E**	98.75	99.38	99.38	100	100	100	100	99.38	98.75	100	**99.56**
	**AB-E**	100	100	100	100	100	100	100	100	100	100	**100**
	**CD-E**	99.58	99.17	100	100	100	100	100	100	99.17	100	**99.79**
	**ABCD-E**	99.75	100	100	100	100	100	100	99.75	100	99.75	**99.93**
**Ternary**	**A-C-E**	99.17	99.58	99.58	100	97.92	98.75	98.75	99.58	100	99.58	**99.29**
	**A-D-E**	100	100	100	100	99.17	100	100	98.75	99.58	100	**99.75**
	**B-C-E**	100	99.58	99.58	100	99.17	99.17	99.17	99.17	99.58	100	**99.54**
	**B-D-E**	99.58	99.17	100	100	100	100	99.58	99.58	100	100	**99.79**
	**AB-CD-E**	99.25	100	99.50	100	99.50	99.00	99.00	99.50	99.00	100	**99.48**
**Five-class**	**A-B-C-D-E**	96.00	94.50	95.50	96.00	95.25	93.50	94.75	96.50	95.00	94.00	**95.10**

**Table 3:** Ablation study results on Bonn five-class classification (%).

Models	Accuracy	Precision	Recall	F_1_
Complete Model	**95.10**	**95.25**	**95.10**	**95.10**
w/o RCM	93.15	93.34	93.15	93.15
w/o DCPM	85.10	87.10	85.10	84.59
w/o Entropy features	94.22	94.37	94.22	94.23

**Table 4:** Impact of different kernel size combinations on five-class classification performance (%).

RCM Kernel Size	DCPM Kernel Size	Accuracy	Precision	Recall	F_1_
3	3	94.00	94.21	94.00	94.00
	5	94.88	95.03	94.88	94.88
	7	94.83	94.94	94.83	94.83
5	3	94.35	94.47	94.35	94.35
	5	**95.10**	**95.25**	**95.10**	**95.10**
	7	94.62	94.63	94.63	94.63
7	3	94.32	94.46	94.32	94.32
	5	94.92	95.04	94.92	94.92
	7	94.40	94.55	94.40	94.40

**Table 5:** Performance comparison between RDPNet and baseline methods on different classification tasks of Bonn dataset.

Dataset		Method	Publication	Acc (%)	RDPNet Acc (%)	RDPNet 95% CI
**Binary**	A-E	ResBiLSTM	Zhao et al., 2024 [38]	**100**	**100**	[100, 100]
		ResNet + LSTM	Qiu et al., 2023 [43]	**100**		
		CNN-Bi-LSTM	Cao et al., 2025 [44]	99.50 ^†^		
	B-E	ResBiLSTM	Zhao et al., 2024 [38]	99.88	99.94	[99.80, 100]
		ResNet + LSTM	Qiu et al., 2023 [43]	**100**		
		CNN-Bi-LSTM	Cao et al., 2025 [44]	98.17 ^†^		
	C-E	ResBiLSTM	Zhao et al., 2024 [38]	**100**	99.88	[99.69, 100]
		ResNet + LSTM	Qiu et al., 2023 [43]	99.78		
		CNN-Bi-LSTM	Cao et al., 2025 [44]	99.75		
	D-E	ResBiLSTM	Zhao et al., 2024 [38]	99.75	99.56	[99.20, 99.93]
		ResNet + LSTM	Qiu et al., 2023 [43]	99.57		
		CNN-Bi-LSTM	Cao et al., 2025 [44]	**100**		
	AB-E	ResBiLSTM	Zhao et al., 2024 [38]	99.92 ^†^	**100**	[100, 100]
		CNN-Bi-LSTM	Cao et al., 2025 [44]	98.60 ^†^		
	CD-E	ResBiLSTM	Zhao et al., 2024 [38]	99.71	**99.79**	[99.54, 100]
		CNN-Bi-LSTM	Cao et al., 2025 [44]	99.11 ^†^		
	ABCD-E	ResBiLSTM	Zhao et al., 2024 [38]	99.83 ^†^	**99.93**	[99.84, 100]
		CNN-Bi-LSTM	Cao et al., 2025 [44]	98.39 ^†^		
**Ternary**	A-C-E	ResBiLSTM	Zhao et al., 2024 [38]	98.88	**99.29**	[98.83, 99.76]
		CNN + LSTM	Shanmugam & Dharmar, 2023 [45]	97.43 ^†^		
	A-D-E	ResBiLSTM	Zhao et al., 2024 [38]	99.04 ^†^	**99.75**	[99.43, 100]
		CNN-Bi-LSTM	Cao et al., 2025 [44]	96.19 ^†^		
		CNN + LSTM	Shanmugam & Dharmar, 2023 [45]	97.36 ^†^		
	B-C-E	ResBiLSTM	Zhao et al., 2024 [38]	99.46	**99.54**	[99.28, 99.80]
		CNN + LSTM	Shanmugam & Dharmar, 2023 [45]	99.09 ^†^		
	B-D-E	ResBiLSTM	Zhao et al., 2024 [38]	99.46 ^†^	**99.79**	[99.58, 100]
		CNN + LSTM	Shanmugam & Dharmar, 2023 [45]	99.37 ^†^		
	AB-CD-E	ResBiLSTM	Zhao et al., 2024 [38]	99.23	**99.48**	[99.18, 99.77]
		ResNet + LSTM	Qiu et al., 2023 [43]	98.17 ^†^		
		CNN-Bi-LSTM	Cao et al., 2025 [44]	95.17 ^†^		
**Five-Class**	A-B-C-D-E	ResBiLSTM	Zhao et al., 2024 [38]	91.27 ^†^	**95.10**	[94.42, 95.78]
		ResNet + LSTM	Qiu et al., 2023 [43]	90.17 ^†^		
		CNN + LSTM	Shanmugam and Dharmar, 2023 [45]	92.50 ^†^		

Note: CI upper bounds exceeding 100% were truncated for interpretability. ^†^ Indicates that the baseline accuracy falls below the lower bound of RDPNet’s 95% CI, suggesting statistical significance at the one-sided α = 0.05 level. The 95% CI refers to intervals computed from ten-fold cross-validation.

**Table 6:** Performance comparison between RDPNet and baseline methods on TUSZ dataset.

Publication	Strategy	Features	Methodology	Acc (%)	F_1,w_ (%)
Li et al., 2020 [46]	CV (5 folds)	Raw EEG	CE-stSENet	92 ^†^	93.69 ^†^
Jia et al., 2022 [47]	CV (5 folds)	Raw EEG	VWCNNs	91.71 ^†^	94 ^†^
Zhang et al., 2022 [48]	CV (10 folds)	VMD	NLTWSVM	92.29 ^†^	92.3 ^†^
Li et al., 2022 [23]	HO (2:1)	FFT	GGN	91 ^†^	91 ^†^
Gill et al., 2024 [49]	HO (4:1)	Multi-domain Feature Set	MHA-CNN	92.1 ^†^	90.2 ^†^
Huang et al., 2023 [50]	CV (5 folds)	Raw EEG	3D-CBAMNet	94.47 ^†^	94.38 ^†^
Zhao et al., 2024 [38]	CV (10 folds)	Raw EEG	ResBiLSTM	95.03 ^†^	95.03 ^†^
RDPNet (Proposed)	CV (5 folds)	Raw EEG	RDPNet	**95.73**	**95.72**

Note: ^†^ Performance lies below RDPNet’s t-based 95% CI (Acc: 95.14–96.32%; F_1,w_: 95.13–96.31%).

**Table 7:** Performance across window sizes on the Bonn dataset in five-class classification.

Window Size (s)	FLOPs (M)	Inference Time (ms)	Accuracy (%)
1	25.86	1.806	88.87
2	51.14	1.817	94.05
3	75.27	1.843	95.10
4	101.74	1.879	94.95

## Data Availability

The datasets generated and/or analyzed during the current study are publicly available from the University of Bonn, Germany. The Bonn EEG epilepsy dataset can be downloaded from the official repository at https://www.ukbonn.de/epileptologie/arbeitsgruppen/ag-lehnertz-neurophysik/downloads/ (accessed on 18 October 2024). The TUSZ dataset is available from Temple University Hospital and can be accessed through the Temple University EEG Corpus at https://isip.piconepress.com/projects/tuh_eeg/ (accessed on 18 October 2024).
